# Persistent increased severity of cannabis use disorder symptoms in adolescents compared to adults: a one-year longitudinal study

**DOI:** 10.1007/s00406-024-01806-y

**Published:** 2024-05-06

**Authors:** Rachel Lees, Will Lawn, Kat Petrilli, Amelia Brown, Katie Trinci, Anya Borissova, Shelan Ofori, Claire Mokrysz, H. Valerie Curran, Lindsey A. Hines, Tom P. Freeman

**Affiliations:** 1 Addiction and Mental Health Group, Department of Psychology, University of Bath, Bath, UK; 2 Department of Psychology, Kings College London, London, UK; 3 Clinical Psychopharmacology Unit, UCL, London, UK; 4 Department of Neuroimaging, Institute of Psychiatry Psychology and Neuroscience, King’s College London, London, UK

**Keywords:** Cannabis use disorder, Adolescence, Longitudinal analysis, CannTeen, Standard THC units

## Abstract

Adolescence is a developmental period characterised by increased vulnerability to cannabis use disorder (CUD). However, previous investigations of this vulnerability have relied on cross-sectional comparisons and lack a detailed assessment of cannabis quantity, a potentially important confounding factor. Here, we aimed to investigate the one-year course of CUD in adolescents compared to adults who currently use cannabis, adjusting for a comprehensive measure of cannabis quantity. Data are from a one-year observational longitudinal study (CannTeen) of adolescents and adults who currently used cannabis regularly with five waves of assessment at 3-monthly intervals, based in London, UK. Participants were *n* = 70 adults (26–29, 45.7% female), who did not regularly use cannabis when they were under age 18, and *n* = 76 adolescents (16–17, 50.0% female). The exposure was adolescent (compared to adult) frequent cannabis use. The primary outcome was CUD symptoms measured using the cannabis use disorder identification test revised (CUDIT-R) at five time points. Models were adjusted for cannabis quantity using mean weekly standard THC units (one unit = 5 mg THC). Other covariates included gender, and whether each session occurred before or during the COVID-19 pandemic. In models adjusted for pre-registered covariates, adolescents scored 3.7 points higher on the CUDIT-R compared to the adult group across the 5 assessment waves (3.66 95% CIs 1.99, 5.34). There was also evidence of a linear reduction in symptoms over time in both groups (−0.47, 95%CIs −0.67, −0.27). Adolescents had persistently increased CUD symptoms compared to adults across the 12-month period. This association was robust after adjusting for the quantity of cannabis consumed and other covariates.

**Supplementary Information:**

The online version contains supplementary material available at 10.1007/s00406-024-01806-y.

## Introduction

Cannabis is used by over 200 million individuals globally, with 4% of the global population reporting use in the past year [1]. This rate is higher in younger people, with 5.8% of 15–16-year-olds reporting past-year use. Regular use of cannabis can lead to a pattern of symptoms causing clinically significant impairment or distress, known as cannabis use disorder (CUD, [2]). DSM-5 criteria for CUD include unsuccessful reduction or quit attempts, cannabis use interfering with daily obligations, worsening mental or physical health, using cannabis in physically hazardous situations, as well as experience of craving, tolerance, and withdrawal symptoms. Early cannabis use is associated with negative outcomes such as poorer mental health and sociodemographic disadvantage in adulthood [3–5]. Furthering our understanding of the consequences of early cannabis use is therefore crucial to trying to reduce the incidence of CUD in adolescence and improve wellbeing during and beyond the adolescent period.

Adolescence is a key developmental period characterised by an increased risk of CUD amongst those who use cannabis. Research has indicated that using cannabis in adolescence is associated with an approximately 3 times increased risk of having CUD compared to using cannabis during adulthood (typically considered as > 18 or > 21; [6–8]), even in samples with the same frequency of use [7, 8]. Further, earlier age-of-onset of cannabis use is associated with an increased risk of having CUD in adulthood [9–13]. However, previous studies comparing adolescent and adult CUD symptoms have had two main limitations. Firstly, evidence for increased risk of CUD in adolescents has typically not accounted for measures of cannabis use. This means that estimates for increased risk of CUD in previous studies may have been inflated due to increased levels of cannabis use in adolescents compared to adults.

The current investigation uses longitudinal data from the CannTeen study, a 12-month observational study developed to provide a direct comparison of adults and adolescents who use cannabis, matched for gender and baseline days per week of cannabis use. Previously published cross-sectional comparisons at baseline replicated previous findings, with a 3.5 times greater risk of severe CUD (measured using DSM-5 criteria) in adolescents, after adjustment for gender, socioeconomic status, risk taking, daily smoking, alcohol use disorder, and other drug use [7].

Adults and adolescents in CannTeen used cannabis at the same frequency at baseline (mean = 4 days per week), but recent research developments have suggested necessary improvements to the measurement of cannabis use [14, 15]. A ‘standard THC unit’ of 5mg THC, the primary psychoactive component of cannabis, has been proposed as a novel measure of cannabis quantity that can be applied to all cannabis products and methods of administration. The standard THC unit has been endorsed by the US National Institutes of Health, and all investigators funded by these institutes are currently required to report research using the standard THC unit in replicable research studies. The standard THC unit can provide rich data on THC dose by incorporating information on potency, frequency, and quantity, all of which are associated with CUD [16–20]. Standard THC units can be assessed using an enhanced cannabis timeline follow-back method (EC-TLFB [21]). The EC-TLFB has been validated in the CannTeen study, with standard THC units showing the strongest validity of all cannabis use measures assessed [21]. It is unclear whether adolescents typically consume more cannabis than adults in an average day of use, which could have influenced their assumed increased vulnerability to CUD in previous studies. Therefore, the current study uses standard THC units as the measurement of cannabis quantity to detect more nuanced differences in profiles of cannabis use across age groups and to identify whether this affects the likelihood of experiencing problems with cannabis use.

Secondly, comparisons have been mostly based on cross-sectional data. Longitudinal analyses are needed to strengthen evidence for the association between age and risk of CUD and to determine the time course of age-related risk. Previous literature has indicated different patterns of CUD that can occur over time, including the adolescent period [21–24]. Studies have reported subgroups who have CUD symptoms that eventually remiss, and subgroups with CUD that increase in severity [21–23]. Younger people may be more likely to have patterns of increasing cannabis use and transition to dependence than older people who use cannabis [25]. To our knowledge, no previous investigations have compared CUD symptoms in adults and adolescents (matched on frequency of cannabis use) longitudinally. Furthermore, the CannTeen study assessed participants at 3-monthly intervals, allowing for a detailed investigation of CUD symptoms as well as cannabis use over a short period which might reduce recall bias and improve accuracy of measurement, and to potentially pick up on shorter-term variation in use.

Here, we present data from a one-year longitudinal study on CUD symptoms in adults and adolescents who use cannabis from the CannTeen dataset. Research questions and hypotheses were pre-registered on the Open Science Framework prior to analyses (https://osf.io/v2afh). We hypothesised that adolescents would show a different pattern of CUD symptoms to adults, characterised by more severe CUD symptoms across the 12 months. We also hypothesised that statistical associations between age, time, and CUD symptoms would be partially attenuated but persist after adjustment for THC units.

## Method

### Participants

Participants were recruited from the London area through social media and Gumtree advertisements, school assemblies, posters, flyers, and word of mouth. Participants met criteria at telephone screening of (1) 1–7 days per week of cannabis use, averaged over the past-3-months, (2) either aged 16–17 or 26–29 years, (3) fluent in English, (4) ability to come to the research facility five times over the upcoming year, (5) normal or corrected-to-normal vision, and (6) capacity to give informed consent. Exclusion criteria included (1) history of diagnosed psychotic episode or disorder, (2) illicit drug use (excluding nitrous oxide) > 2 times per month, over the past-3-months, (3) nitrous oxide use > 1 day per week over the past-3-months, (4) receiving of treatment for any mental health condition (including CUD) in the past month, (5) currently daily use of a medication which is commonly psychotropic, (6) any mental or physical health condition deemed problematic by a medical doctor, and (7) age-adjusted body mass index (BMI) < 2nd or > 99.6th percentile. An additional exclusion criterion for the adult group was cannabis use at a frequency of once per week or more (averaged over a 3 month or longer period) before the age of 18.

Age ranges were chosen as the earliest time point at which adolescents do not require parental consent to take part in a research study in the UK (age 16), and for adults after the age at which adolescent brain development is generally complete (> 25 years, [26]). Inclusion criteria for cannabis frequency ensured that the participants were at least weekly users of cannabis. Finally, the criteria for no regular use of cannabis under age 18 in the adult group in CannTeen was chosen to isolate the effects of frequent cannabis use in adolescence (the adolescent group) on relevant outcomes, compared to a group that did not have this exposure (the adult group).

### Procedures and measures

At the baseline visit, BMI was confirmed to be within the specified limits, and a valid form of ID was used to confirm participants’ current age. At all sessions, participants confirmed absence from alcohol and cannabis use for the previous 12 h, and other illicit drug use for 48 h via self-report, saliva drug screening, and breathalyser testing. Sessions took place at the Clinical Psychopharmacology Unit, UCL, central London. Testing sessions took place 3 months apart, with participants encouraged to attend as close to this schedule as possible but permitted to attend up to 2 weeks early and 6 weeks late if necessary. The CannTeen study ran from November 2017 to June 2021. Sessions after 23rd March 2020 had to be adapted to virtual data collection during the national COVID-19 lockdown periods in the UK. Virtual research sessions retained as many of the features of in-person data collection as possible. The assessments pertaining to this manuscript were not meaningfully altered by this change in data collection. However, we were unable to objectively determine the absence of recent alcohol, cannabis, or other drug use virtually and, therefore, these eligibility criteria were only fulfilled using self-report.

### Analysis variables

#### Outcome

Participants completed the Cannabis Use Disorder Screening Test Revised (CUDIT-R; [27]) at each of the five testing sessions. This self-report measure assessed past-3-month symptoms related to cannabis use, including items related to frequency of use, duration of time spent ‘stoned’ on a typical day, difficulty stopping use, failing to meet obligations due to use, spending a lot of time on cannabis, problems with memory or concentration after using, using in situations that could be physically hazardous, and whether they had thought about stopping or reducing their use. The frequency that each of the 8 symptoms had occurred (never, less than monthly, weekly, daily, or almost daily) was recorded, and a numerical score was assigned to each. The CUDIT-R has good internal consistency and concurrent validity [28]. Total scores for the CUDIT-R range from 0 to 32.

#### Predictor

The predictor variable in this analysis was age group: adolescent (16–17 years) vs adult (26–29) years.

#### Covariates

##### Measure of cannabis use—standard THC units

The EC-TLFB [21] was used to estimate mean weekly standard THC unit consumption at each time point. Participants provided details on all cannabis *types* used in the past 3 months (sinsemilla, hash/resin, seeded herbal, other), and all *methods* used (e.g., joint, bong, pipe, vaporiser, and ingested). They gave estimates of the number of grams of cannabis typically used with each method and indicated how much they would normally use of the method from a scale of 1–10. A 3-month TLFB was then completed with the participant at each session, noting every occasion of each method of cannabis use over the time period.

To approximate potency for the three main cannabis types reported in CannTeen we used estimates from UK seizure data. From the most recent available data [29], the estimates were 14.2% for sinsemilla ‘skunk’ type cannabis; 6.3% for hash/resin, and 3.5% for seeded-herbal cannabis. Some CannTeen participants reported the use of other cannabis types, including shatter/wax, THC oil and trichome powder (‘kief’). For these types, where appropriate, we again used estimates from Potter et al., 2018 (shatter/wax 78%, THC oil 51%, and trichome powder 40.15%). These were based on notably fewer samples than the main cannabis types, reflecting their less common use in the UK population. See Online Resource 1 for more details on how standard THC units were estimated in the presence of missing data.

##### COVID-19 time-period indicator

To adjust for the CannTeen study running during the COVID-19 pandemic and subsequent lockdown periods, a binary variable indicating whether each session occurred before 23rd March 2020 (the date of the first nationwide lockdown in the UK) as (0) or after (1) was entered into adjusted models as a covariate.

##### Gender

Participant gender was added as a covariate in adjusted models due to evidence indicating gender differences in the risk of CUD [30]. Participants were asked to report their gender at screening (“male”, “female”, “other”), participants only reported gender categories of “male” and female”.

##### Mental health

At each testing session, symptoms of anxiety and depression were assessed using the Beck Anxiety Inventory and Beck Depression Inventory, respectively. Total scores range from 0 to 63 on both measures. Exploratory analyses include these as time-varying covariates.

##### Other drug use

Detailed assessments of drug use were conducted using TLFB methodology. In line with other CannTeen investigations [7], exploratory analyses adjusted for daily cigarette smoking, alcohol use on two or more days per week, and other illicit drug use on 1 or more days per month.

### Statistical analysis

Before analysing the data, we pre-registered the predictor, covariate, and outcome variables for this analysis on the Open Science Framework (https://osf.io/v2afh). The effect of age group (adolescent vs adult) on CUDIT-R score over time was analysed using linear mixed-effects models, using the “lme4” package in R. Multi-level modelling of longitudinal data allows for adjustment of within-person variation due to repeated measurements from the same individual not being independent. These models also allow for the exploration of the effect of predictors on the outcome, accounting for the clustering of data across the repeated measurements. The outcome variable in all models was the CUDIT-R score, and all models included a random intercept of participant. Fixed effects included age group, time, and age*time interaction. The interaction term was included based on the assumption that cannabis use would continue over the year period and to assess whether this would involve a worsening of CUD symptoms in the adolescents compared to the adults. A quadratic fixed effect of time was assessed and did not improve model fit, so was not retained in subsequent models. Age group was a binary variable, coded using Helmert coding, thus the regression coefficient can be interpreted as the mean difference in CUDIT-R scores between adult and adolescent groups. Adjusted versions of the model included a time-invariant fixed effect of gender (0 = male, 1 = female), and time-varying fixed effects of the COVID-19 pandemic indicator and weekly standard THC units. Additional exploratory sensitivity analysis included all previous covariates as well as adding mental health, tobacco, alcohol, and other drug use as time-varying covariates. Model fit was compared using the Akaike information criterion (AIC), Bayesian information criterion (BIC), and −2 log likelihood (-2LL).

Mean weekly standard THC unit data were winsorized at 95% and 5% quantiles, using the R package “Winzorise”, to minimise bias from outlying or implausible estimates. This method involves replacing values that lie above or below the 95% and 5% percentiles (respectively) with the values at these percentile limits. Sensitivity analyses indicated that model estimates were very similar when using standard THC unit data with and without winsorizing (see Online Resource, Table 6). A power calculation was conducted to detect cross-sectional differences in CUD by age group (reported in [7]), based on previous studies indicating an odds ratio of 3 [9–11]. This indicated 148 participants were required, split evenly by age group.

As mixed effects models use maximum likelihood estimation, all participants were included in the analysis, despite participants contributing a different amount of data due to dropout or missing sessions (see Online Resource, Table 2). Therefore, there was no need to use multiple imputation or other accounting for missing data in this analysis. However, there was some very minimal (*n* = 3) missing data in the exploratory sensitivity analysis. Participants with complete CUDIT-R data did not differ from those missing any CUDIT-R data on gender (p = 0.722), CUDIT-R baseline total scores (p = 0.346), depression (p = 0.142), anxiety (p = 0.751), daily smoking (p = 0.540), > 2 weekly alcohol use (p = 0.263), or > 1 per month other drug use at baseline (p = 0.600). Age groups did not differ on mean number of sessions with available CUDIT-R data (adolescents: 4.20, adults: 4.00, p = 0.384). See Online Resource, Tables 2 and 3 for further details. We therefore did not have concerns about bias related to missing data influencing our model outcomes (Online Resource, Tables 1, 2).

**Table 1:** Baseline sample characteristics. Data shown are frequencies and means (standard deviations) as appropriate

	Adolescent	Adult
*n*	76	70
Age range	16–17	26–29
Gender (%Female)	50.0	45.7
Ethnicity		
% White	68.0	64.3
% Asian	2.7	15.7
% Mixed	20.0	10.0
% Black	5.3	8.6
% Other	4.0	1.4
Socioeconomic status		
% Maternal education undergraduate degree or above	58.67	44.93
% Maternal education below undergraduate degree	41.33	55.10
Days per week cannabis use	3.78 (2.0)	4.21 (1.9)
Mean age of first cannabis use	14.6 (1.1)	18.0 (2.9)
% Daily cigarette smoking	13.16	12.86
% Alcohol use > = 2 days per week	2.63	28.57
% Other drug use > = one day per month	59.21	25.71
Depression	12.71 (8.34)	7.90 (8.83)
Anxiety	12.49 (10.12)	7.62 (7.57)

Depression and Anxiety scores are from the Beck Depression and Beck Anxiety Inventories. Means/frequencies calculated from available data

**Table 2:** Mean standard THC units at each time point, by age group

Time point	Age group	
	Adolescent Mean (sd)	Adult Mean (sd)
Baseline	77.23 (82.55)	65.61 (83.09)
3 months	65.51(71.55)	66.57 (86.91)
6 months	64.12 (76.75)	58.40 (76.63)
9 months	73.20 (84.87)	58.21 (84.67)
12 months	72.84 (83.33)	57.40 (82.13)

Standard THC units were winsorized at 95% and 5% quantiles. Data are means from available data, missing data varies across time points

## Results

### Model outcomes

**Fig. 1:**
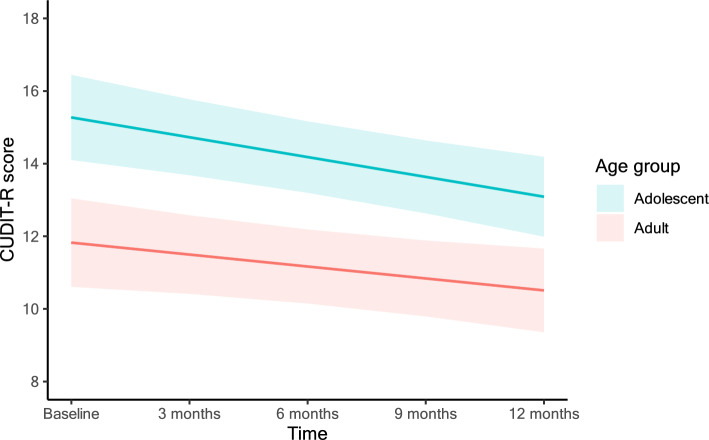
Model estimated means of CUDIT-R scores at each time point in the fully adjusted* model, stratified by age. *covariates included gender, whether each session occurred before or during the COVID-19 pandemic and mean weekly standard THC units. Error bars display 95% confidence intervals

The intraclass correlation in the fully adjusted model was 0.50. Age group was associated with CUDIT-R-score, with adolescents scoring on average 3.7 points higher on the CUDIT-R than adults across the 5 assessment waves (3.68, 95%CIs 1.81, 5.56). A linear decrease in CUDIT-R scores over time was observed (−0.47, 95%CIs −0.67, −0.27). There was a lack of evidence for a time by age interaction (−0.10, 95%CIs −0.49, 0.30). Adjusting for covariates of gender, COVID-19 and standard mean weekly THC units did not alter this pattern of results, with adolescents scoring 3.7 points higher (3.66, 95%CIs 1.99, 5.34) compared to adults. Standard THC units and BDI scores were the only covariates with evidence of an independent effect on CUDIT-R scores. See Fig. 1 for model estimates of CUDIT-R scores over the 5 time points (Tables 3, 4).

**Table 3:** Model estimates of the effect of age time, and age by time interaction on CUDIT-R scores

	Unadjusted B (95%CI), *p* *n* = 146		Adjusted^1^ B (95%CI), *p* *n* = 146		Adjusted^2^ B (95%CI), *p* *n* = 146		Sensitivity Adjusted^3^ B (95%CI), *p* *n* = 143	
Age								
Adolescent vs Adult	3.68 (1.81, 5.56)	< 0.001	3.78 (1.92, 5.64)	< 0.001	3.66 (1.99, 5.34)	< 0.001	3.12 (1.37, 4.86)	< 0.001
Time	−0.47 (−0.67, −0.27)	< 0.001	−0.47 (−0.70, −0.25)	< 0.001	−0.44 (−0.66, −0.22)	< 0.001	−0.46 (−0.69, −0.23)	< 0.001
Age*Time interaction								
Adolescent vs Adult	−0.10 (−0.49, 0.30)	0.629	−0.15 (−0.55, 0.25)	0.464	−0.22 (−0.61, 0.18)	0.279	−0.23 (−0.64, 0.18)	0.264
Gender (Male vs Female)	–		0.72 (−0.79, 2.23)	0.352	0.53 (−0.75, 1.81)	0.417	1.10 (−0.16, 2.36)	0.088
COVID-19 status	–		−0.00 (−1.19, 1.19)	0.997	−0.16 (−1.32, 1.00)	0.790	0.02 (−1.14, 1.18)	0.976
Mean weekly standard THC units	–		–		0.02 (0.02, 0.03)	< 0.001	0.02 (0.02, 0.03)	< 0.001
Anxiety	–		–		–		0.01 (−0.06, 0.07)	.784
Depression	–		–		–		0.12 (0.05, 0.18)	< 0.001
Daily cigarette use	–		–		–		0.82 (−0.39, 2.02)	0.183
Alcohol use > = 2 days weekly	–		–		–		−0.69 (−1.83, 0.44)	0.231
Other illicit drug use (≥ one day per month	–		–		–		0.08 (−0.76, 0.91)	0.851
AIC	3487.45		3356.16		3303.48		3187.94	
BIC	3513.83		3391.02		3342.69		3248.43	
-2 LL	−1737.73		−1670.08		−1642.74		−1579.97	

^1^Adjusted for time-invariant covariate of gender and time-varying covariate of COVID-19 status at the time of session

^2^Adjusted for time-invariant covariate of gender and time-varying covariates of COVID-19 status at the time of session, and mean weekly standard THC units

^3^Adjusted for time-invariant covariate of gender, and time-varying covariates of COVID-19 status at the time of session, mean weekly standard THC units, Beck Anxiety Inventory Score, Beck Depression Inventory Score, daily cigarette smoking, alcohol use (≥ 2 days weekly), and other illicit drug use (≥ one day per month)

**Table 4:** Comparison of CUDIT-R items endorsed, by age group at baseline assessment

CUDIT-R items	Adolescent item endorsed *n* (%)	Adult item endorsed *n* (%)	Comparison of adolescent vs Adult: Odds Ratio (*p* value)
Q1. Use of cannabis	76 (100)	70 (100)	–
Q2. 1 or more hours stoned on a typical day using cannabis	75 (98.68)	66 (94.29)	4.55 (*p* = 0.181)
Q3. Not able to stop using cannabis once started	36 (47.37)	^1^19 (27.54)	2.37 (*p* = .015)
Q4. Failed to do what was normally expected because of using cannabis	54 (71.05)	14 (20.00)	9.82 (*p* < 0.001)
Q5. Devoted a great deal of time to getting, using or recovering from cannabis	62 (81.58)	34 (48.57)	4.69 (*p* < 0.001)
Q6. Problem with memory or concentration after using cannabis	68 (89.47)	^1^50 (72.46)	3.23 (*p* = 0.011)
Q7. Use of cannabis in situations that could be physically hazardous^2^	12 (15.79)	^1^13 (18.84)	0.81 (*p* = 0.628)
Q8. Thought about cutting down, or stopping, cannabis use	67 (88.16)	56 (80.00)	1.86 (*p* = 0.181)

^1^missing *n* = 1

^2^examples of physically hazardous situations include driving, operating machinery, or caring for children

### Exploratory sensitivity analysis

To account for potential confounding of mental health, and other drug use (including alcohol, tobacco, and other illicit drugs), we ran an exploratory analysis adding these into the fully adjusted main model (see Table 3). This did not substantially alter the pattern of results, with a main effect of age (3.12, 95%CIs 1.37, 4.86), and a general linear decrease over time, (−0.46, 95%CIs −0.69, −0.23), with no age by time interaction (−0.23, 95%CIs −0.64, 0.18).

We also ran a sensitivity analysis using quartiles of total THC consumption as the measure of cannabis use, which did not change the overall model findings, see Online Resource, Table 4 for full model outcomes.

## Discussion

In this one-year, longitudinal investigation of adolescents and adults who use cannabis, we found that adolescents (aged 16–17) scored on average 3.7 points higher on the CUDIT-R than adults (aged 26–29) across all 5 assessment waves (3.68, 95% CIs 1.81, 5.56). This effect was only partially attenuated after adjustment for gender, COVID-19, and mean weekly standard THC units (3.66 95% CIs 1.99, 5.34). CUD symptoms decreased linearly over the year in both age groups (−0.47, 95%CIs −0.67, −0.27). Through the use of a longitudinal study with five assessment waves, and a comprehensive standardised assessment of cannabis exposure, these findings show that the increased number of CUD symptoms that have been observed in adolescents persists over 12 months and is robust after adjustment for variation in THC dose.

Evidence of the persistence of increased levels of CUD symptoms in adolescents compared to adults across the 12-month period builds on previous cross-sectional comparisons of the likelihood of CUD by current age [6–8, 29, 30]. To our knowledge, the current study is the first such longitudinal comparison of adult and adolescent symptoms. This study is important because longitudinal comparisons can provide higher quality of evidence than cross-sectional comparisons. Furthermore, they can provide insight into the time course of such associations. The findings indicate that this is a persistent effect over a year, highlighting the need for a comprehensive evaluation of the impact of cannabis use on adolescent health and wellbeing. Adolescents often endorsed items related to cannabis affecting their general functioning, including failing to meet obligations and dedicating a lot of time to cannabis use. This indicates that the use of cannabis at this age has the potential to disrupt adolescents’ personal or academic lives, which could result in difficulties with educational outcomes and transitions into adulthood [31]. Given these findings, it is crucial that appropriate healthcare resources are available for this age group; however, the transition from child (< 18) to adult (> 18) health services can be challenging, and there is a risk of young people falling through the cracks [32]. Our findings add weight to the idea of integrated young peoples’ services covering a wider age range (e.g., 12 to 25). Further avenues of support could include education and harm reduction advice tailored for young people, as well as public health/policy-related changes to reduce stigma and barriers related to treatment seeking for cannabis-related support and increasing accessibility of support for young people [33]. Additionally, there was a linear decrease in CUD symptoms over time in both groups. This could be an indication of ‘maturing out’ from CUD [34]. However, longer follow-up periods would be necessary to demonstrate robust changes in CUD symptoms such as long-term remission. Furthermore, group means on the CUDIT-R were still elevated in both groups at the 12-month follow-up. By including longer follow-ups, such studies could provide valuable insight into the course of adolescent risk of CUD.

Some previous investigations have used samples with matched or similar levels of cannabis frequency [7, 8]; however, most do not consider cannabis quantity. Cannabis use profile (including frequency, quantity, and potency of use) has been consistently linked to the risk of CUD [18–20], and adolescents may use higher quantities of cannabis than adults. Therefore, not accounting for this could have led to overinflation of estimates of risk in adolescents. The current analysis used a novel measurement of cannabis quantity, the THC unit (5mg THC). Here, we found that adolescents did report greater cannabis use using this measure that incorporates quantity, frequency, and potency. However, adjusting for this in models did not substantially alter the main effect of a greater number of symptoms in adolescents, indicating that increased cannabis use in adolescents was not primarily responsible for the increased CUD symptoms.

The current analysis investigated two important factors related to CUD: current age, and profile of cannabis use. However, there are several other factors that might influence the relationship between cannabis use during adolescence and CUD symptoms that were not accounted for in the main analysis model. For example, CUD often co-occurs with other mental health disorders and other substance use disorders [31, 35]. We chose not to adjust for this in the primary analysis due to concerns over the direction of causality, given that other mental health disorders could either act as a mediator of the relationship between adolescent cannabis use and CUD symptoms or as a common cause of both [36]. However, in an exploratory sensitivity analysis, we added mental health and other drug use to the model, and adolescents still scored on average 3.1 points higher on the CUDIT-R than adults. This provides more support for the role of adolescent vulnerability to CUD, as this will account for more relevant confounders. However, other factors could still confound the relationship between adolescent frequent cannabis use and CUD [37]. For example, we were unable to account for genetic risk factors that may have differed between the adolescent and adult groups. Further studies with larger sample sizes will be needed to provide adequate power to adjust for a more comprehensive set of potential confounds, to increase precision when estimating the risk of CUD in adolescents compared to adults.

Strengths of this study include its longitudinal design with five assessment waves, the use of a validated outcome variable (CUDIT-R score), and a comprehensive standardised assessment of THC exposure validated in this sample [21]. The current findings should be considered in the light of several limitations. Firstly, the study was limited to only a 12-month follow-up duration, restricting the conclusions that can be drawn about longer-term CUD across adolescence and into adulthood. Furthermore, the measurement of CUD symptoms was the CUDIT-R, rather than the diagnostic DSM-5 clinical interview. However, at the baseline assessment, mean CUDIT-R scores increased across DSM-5 severity classifications (see Online Resource, Fig. 1). The CUDIT-R has not been validated for use over periods shorter than 6 months and, therefore, this may have induced unintended consequences. For example, it could be that assessment over a shorter period of time influences cannabis use in some way. However, there was no indication that this had a different effect on either age group given the lack of time by age interaction on CUDIT-R scores. Our finding of reduced CUDIT-R symptoms across groups could be in part due to the influence of being part of this longitudinal study. Repeated assessment of the CUDIT-R as well as administration of the TLFB to assess drug use may have in some way acted as an intervention (e.g., due to increased self-monitoring of drug use), bringing participants’ attention to their cannabis use and encouraging reduction of use. Another consideration is whether instruments assessing CUD symptoms are appropriate for comparison across age groups. The CUDIT-R has been implemented in adolescent samples previously; however, little research has investigated measurement invariance of CUD assessments, a key assumption underlying comparison of age groups, and therefore this should be considered a necessary avenue for future research into adolescent/adult comparisons.

Additionally, whilst standard THC units can estimate the dose for all cannabis products and methods of administration, they may be influenced by participant error in reporting (e.g., estimation of grams). However, estimated standard THC units using these methods were associated with objectively verified THC exposure (THC:COOH/creatinine) with a large effect size (r = 0.52), with a stronger correlation than any other measure of cannabis use from the CannTeen dataset [21]). Our estimates of cannabis potency were based on available UK seizure data [29]. Cannabinoid potency testing can potentially be biased due to degradation in sample quality prior to testing for cannabinoid testing, which could lead to underestimates of the recorded THC concentration [38]. However, in the investigation of UK seizure data researchers found that CBN concentrations were low in their samples and this was not related to the length of time the samples were in storage for, with the authors indicating that these samples were a fair representation of the original seized materials [29].

Additionally, the sample size was modest and not sampled to be representative of the general population of people who use cannabis due to inclusion criteria around use. This enabled purposeful sampling of matched groups of adolescents and adults who use cannabis at the same high, mean frequency to increase the meaningfulness of comparisons. This approach can be considered advantageous to population cohort studies, as the prevalence of regular adolescent cannabis use in the general population is rare resulting in small sample sizes. Additionally, greater levels of cannabis use in adolescence than in adulthood could lead to overestimates of adolescent risk. Therefore, purposively sampling matched adult and adolescent groups can overcome these limitations. However, because of this sampling approach, these findings may not be representative of CUD risk in people who use cannabis less than weekly.

Criteria for the adult group to have had minimal cannabis use under age 18 means that they are likely not representative of the average adult who uses cannabis and are likely to differ from the adolescent group on other variables related to CUD. This design was chosen to isolate the effects of adolescent cannabis use compared to those from adult use, to investigate whether cannabis is associated with more harm when used frequently in adolescence. Given our inclusion criteria for adult participants to have no regular use of cannabis before the age of 18, our adult and adolescent groups varied based on their reported age of first cannabis use. As a sensitivity analysis, we included the age of first use as a covariate in the models, which did not substantially alter the pattern of results (see Online Resource, Table 5). Furthermore, the CannTeen sample was limited to those in the London area, and those willing to take part in a study with relatively frequent assessments and therefore high levels of engagement. The findings from this study should be viewed in the light of this.

These findings add to a wider literature on adolescent vulnerability to CUD, predominantly comprised of large-scale surveys. These research designs tend to have good statistical power to adjust for important confounding factors, but they typically lack detailed data on participants’ cannabis use and are mostly cross sectional. The current study therefore adds to the literature by examining the one-year course of CUD symptoms, adjusting for a detailed assessment of cannabis use and other relevant covariates. However, the clinical meaning of the current observed difference in CUDIT-R scores is yet to be determined, as we are not aware of work that has assessed the clinical meaning of CUDIT-R scores. Lived experience feedback from people who use and support those who use cannabis, including from adolescents themselves, is needed to further establish the implications of these findings.

In conclusion, the current study provides the first evidence of longitudinal persistence of increased severity of CUD in adolescents compared to adults, with adolescents on average scoring 3.7 points higher on a measure of CUD symptoms, over one year. This pattern of results remained after adjustment for a comprehensive measure of cannabis quantity. This study indicates the increased risk of CUD symptoms in adolescents and provides evidence to support the importance of delaying or minimising the use of cannabis during this developmental period.

## Supplementary Information

Below is the link to the electronic supplementary material.

Supplementary file1 (DOCX 44 kb)

## Data Availability

The participants of this study did not give written consent for their data to be shared publicly, so the research supporting data are not available.
