# Supplementary material for: Persistent increased severity of cannabis use disorder symptoms in adolescents compared to adults: a one-year longitudinal study
Source: Eur Arch Psychiatry Clin Neurosci. 2024 May 6;275(2):397–406. doi: 10.1007/s00406-024-01806-y (PMC11910442; doi:10.1007/s00406-024-01806-y)
Supplement: Supplementary file 1 — Supplementary file1 (DOCX 44 kb) [file 406_2024_1806_MOESM1_ESM.docx]

**Persistent increased severity of cannabis use disorder symptoms in adolescents compared to adults: a one-year longitudinal study**

# Online Resource 1

Rachel Lees^1^, Will Lawn^2^, Kat Petrilli^1^, Amelia Brown^1^, Katie Trinci^3^, Anya Borissova^3,4^, Shelan Ofori^3^, Claire Mokrysz^3^, H Valerie Curran^3^, Lindsey A Hines^1^, Tom P Freeman^1^

^1^Addiction and Mental Health group, Department of Psychology, University of Bath

^2^Department of Psychology, Kings College London

^3^Clinical Psychopharmacology Unit, UCL

^4^Department of Neuroimaging, Institute of Psychiatry Psychology and Neuroscience, King’s College London, UK

**Corresponding author**

Rachel Lees, rhl32@bath.ac.uk

**Estimating calculating standard THC unit data in presence of missing data**

Participants were able to estimate the quantity of cannabis they had used in each method in the vast majority of cases. Occasionally, participants found it difficult or refused to estimate the grams of cannabis they used in a particular method. This occurred for methods such as edibles (including brownies, cookies, cake, chocolate, butter and sweets), where the participant may not have seen the amount of cannabis added to the method. In the first instance, if the participant had used this method at a different occasion and provided an estimate of quantity, we substituted this value for the missing value. In cases where the participant did not have other data to estimate from, we used average data from the whole CannTeen sample for each type. Median quantities of different edible types are found in Table 1.

Table 1. Estimates of cannabis quantity across types of ‘edibles’

| Cannabis edible type | Median quantity (g) from whole sample |
| --- | --- |
| Sinsemilla cannabis in a brownie/cookie/cake | 0.45 |
| Sinsemilla cannabis in chocolate | 0.1 |
| Sinsemilla cannabis in butter | 0.5 |
| Sinsemilla cannabis in a sweet/gummy | 0.075 |

Participants also struggled to estimate quantity when reporting use of THC oil in a vaporiser. When data was provided for this method, it tended to be inconsistent, with some participants providing data on the quantity in a cartridge but estimating their uses in terms of ‘puffs’ or giving implausible estimates for one occasion of use (e.g., 20ml), that varied from other occasions. Because of this, I decided that using the whole group median (0.3g) was inappropriate, as doing so would very likely lead to overestimation, particularly in cases when use was reported as only one or two puffs. Instead, for all occasions that a participant reported THC use in a vaporiser, we estimated quantity by calculating the mean of the other cannabis administration methods used by the participant at that occasion, and substituted potency for the mean of the main three cannabis types (8%). One CannTeen participant used THC oil in a vaporiser as their primary method of use on several occasions, with inconsistent quantity estimates and sometimes only one other method of use in which to estimate from. I decided that estimating quantity and potency of use for this participant was problematic, and their data was not used in this analysis.

Finally, a small number of participants reported use of trichome powder ‘kief’ added on top of a sinsemilla joint but did not estimate the quantity, describing this as a ‘sprinkle’ and the amount as ‘negligible’. We consulted with a cannabis user who uses this method to get an estimate of the grams in a ‘sprinkle’ of trichome powder added to a joint and they estimated this to be 0.01 grams, which was substituted for the quantity in two cases.

Table 2. Missing data on total CUDIT score at each time point, separately by age group

| Time point | Age group | |
| --- | --- | --- |
|  | Adolescent  *N* (%) | Adult  *N* (%) |
| Baseline | 0 (0) | 2 (2.82) |
| 3-month | 11 (14.47) | 12 (16.90) |
| 6-month | 16 (21.01) | 22 (31.00) |
| 9-month | 20 (26.32) | 20 (28.17) |
| 12-month | 14 (18.42) | 14 (19.72) |

*Note that missing data is handled in analysis models via maximum likelihood, and no participants were removed from the analysis due to missing CUDIT data.

Table 3. Amount of available data on CUDIT-R, by age group

|  | Number of sessions with complete CUDIT-R data | | | | |
| --- | --- | --- | --- | --- | --- |
|  | 1  N(%) | 2  N(%) | 3  N(%) | 4  N(%) | 5  N(%) |
| Adolescent | 8 (10.53) | 4 (5.26) | 4 (5.26) | 9 (11.84) | 51 (67.11) |
| Adult | 7 (10.00) | 4 (5.71) | 10 (14.29) | 10 (14.29) | 39 (55.71) |

Table 4. Model estimates of the effect of age time, and age by time interaction on CUDIT-R scores, when using a measure of quartiles of standard THC units

|  | Unadjusted  B (95%CI), *p*  *N* = 146 | | Adjusted^1^  B (95%CI), *p*  *N* = 146 | | Adjusted^2^  B (95%CI), *p*  *N* = 146 | | Sensitivity Adjusted^3^  B (95%CI), *p*  *N* = 143 | |
| --- | --- | --- | --- | --- | --- | --- | --- | --- |
| **Age** |  | |  | |  | |  | |
| Adolescent vs Adult | 3.68 (1.81, 5.56) | <.001 | 3.78 (1.92, 5.64) | <.001 | 3.01 (1.40, 4.62) | <.001 | 2.62 (0.94, 4.29) | .002 |
| **Time** | -0.47 (-0.67, -0.27) | <.001 | -0.47 (-0.70, -0.25) | <.001 | -0.46 (-0.67, -0.25) | <.001 | -0.48 (-0.70, -0.26) | <.001 |
| Age*Time interaction |  | |  | |  | |  | |
| Adolescent vs Adult | -0.10 (-0.49, 0.30) | .629 | -0.15 (-0.55, 0.25) | .464 | -0.12 (-0.50, 0.26) | .525 | -0.15 (-0.54, 0.24) | .445 |
| Gender (Male vs female) | - | | 0.72 (-0.79, 2.23) | .352 | 0.07 (-1.17, 1.30) | .917 | 0.57 (-0.64, 1.78) | .356 |
| COVID-19 status | - | | -0.00 (-1.19, 1.19) | .997 | -0.32 (-1.44, 0.79) | .570 | -0.16 (-1.28, 0.96) | .783 |
| Quartile of THC consumed | - | | - | | 2.07 (1.70, 2.43) | <.001 | 1.97 (1.60, 2.34) | <.001 |
| Anxiety | - | | - | | - | | -0.00 (-0.06, 0.06) | .987 |
| Depression | - | | - | | - | | 0.12 (0.05, 0.18) | <.001 |
| Daily cigarette use | - | | - | | - | | 0.53 (-0.63, 1.69) | .374 |
| Alcohol use >=2 days weekly | - | | - | | - | | -0.54 (-1.63, 0.55) | .327 |
| Other illicit drug use (>=one day per month | - | | - | | - | | 0.13 (-0.67, 0.93) | .752 |

^1^Adjusted for time-invariant covariate of gender and time-varying covariate of COVID-19 status at time of session

^2^ Adjusted for time-invariant covariate of gender and time-varying covariates of COVID-19 status at time of session, and mean weekly standard THC units

^3^ Adjusted for time-invariant covariate of gender, and time-varying covariates of COVID-19 status at time of session, mean weekly standard THC units, Beck Anxiety inventory score, Beck Depression inventory score, daily cigarette smoking, alcohol use (>=2 days weekly), and other illicit drug use (>=one day per month)

Table 5. Model estimates of the effect of age time, and age by time interaction on CUDIT-R scores with age of first use added as a covariate

|  | Unadjusted  B (95%CI), *p*  *N* = 146 | | Adjusted^1^  B (95%CI), *p*  *N* = 146 | | Adjusted^2^  B (95%CI), *p*  *N* = 146 | | Sensitivity Adjusted^3^  B (95%CI), *p*  *N* = 143 | |
| --- | --- | --- | --- | --- | --- | --- | --- | --- |
| **Age** |  | |  | |  | |  | |
| Adolescent vs Adult | 3.10 (0.87, 5.33) | .007 | 3.25 (1.04, 5.46) | .004 | 3.66 (1.70, 5.62) | <.001 | 2.98 (0.95, 5.01) | .004 |
| **Time** | -0.47 (-0.67, -0.27) | <.001 | -0.47 (-0.70, -0.25) | <.001 | -0.44 (-0.66, -0.22) | <.001 | -0.46 (-0.69, -0.23) | <.001 |
| Age*Time interaction |  | |  | |  | |  | |
| Adolescent vs Adult | -0.09 (-0.49, 0.30) | .642 | -0.15 (-0.55, 0.25) | .474 | -0.22 (-0.61, 0.18) | .280 | -0.23 (-0.64, 0.18) | .268 |
| Age of first cannabis use | -0.01 (-0.04, 0.02) | .340 | -0.01 (-0.04, 0.02) | .390 | -0.00 (-0.03, 0.03) | .993 | -0.00 (-0.03, 0.02) | .799 |
| Gender (Male vs female) | - | | 0.70 (-0.81, 2.21) | .361 | 0.53 (-0.76, 1.82) | .419 | 1.10 (-0.17, 2.36) | .090 |
| COVID-19 status | - | | -0.02 (-1.22, 1.17) | .968 | -0.16 (-1.32, 1.00) | .788 | 0.01 (-1.16, 1.17) | .989 |
| Mean weekly standard THC units | - | | - | | 0.02 (0.02, 0.03) | <.001 | 0.02 (0.02, 0.03) | <.001 |
| Anxiety | - | | - | | - | | 0.01 (-0.06, 0.07) | .786 |
| Depression | - | | - | | - | | 0.12 (0.05, 0.18) | <.001 |
| Daily cigarette use | - | | - | | - | | 0.82 (-0.39, 2.02) | .184 |
| Alcohol use >=2 days weekly | - | | - | | - | | -0.71 (-1.85, 0.43) | .224 |
| Other illicit drug use (>=one day per month | - | | - | | - | | 0.07 (-0.76, 0.91) | .861 |

Table 6. Model estimates of the effect of age time, and age by time interaction on CUDIT-R scores with standard THC unit variable not winsorized

|  | Unadjusted  B (95%CI), *p*  *N* = 146 | | Adjusted^1^  B (95%CI), *p*  *N* = 146 | | Adjusted^2^  B (95%CI), *p*  *N* = 146 | | Sensitivity Adjusted^3^  B (95%CI), *p*  *N* = 143 | |
| --- | --- | --- | --- | --- | --- | --- | --- | --- |
| **Age** |  | |  | |  | |  | |
| Adolescent vs Adult | 3.68 (1.81, 5.56) | <.001 | 3.78 (1.92, 5.64) | <.001 | 3.82 (2.05, 5.59) | <.001 | 3.14 (1.31, 4.98) | <.001 |
| **Time** | -0.47 (-0.67, -0.27) | <.001 | -0.47 (-0.70, -0.25) | <.001 | -0.46 (-0.68, -0.23) | <.001 | -0.48 (-0.71, -0.25) | <.001 |
| Age*Time interaction |  | |  | |  | |  | |
| Adolescent vs Adult | -0.10 (-0.49, 0.30) | .629 | -0.15 (-0.55, 0.25) | .464 | -0.21 (-0.61, 0.20) | .315 | -0.22 (-0.64, 0.19) | .001 |
| Gender (Male vs female) | - | | 0.72 (-0.79, 2.23) | .352 | 0.64 (-0.76, 2.04) | .368 | 1.26 (-0.10, 2.63) | .070 |
| COVID-19 status | - | | -0.00 (-1.19, 1.19) | .997 | .997 | .771 | 0.00 (-1.19, 1.19) | .998 |
| Mean weekly standard THC units | - | | - | | 0.01 (0.01, 0.01) | <.001 | 0.01 (0.01, 0.01) | <.001 |
| Anxiety | - | | - | | - | | 0.01 (-0.06, 0.08) | .749 |
| Depression | - | | - | | - | | 0.13 (0.06, 0.20) | <.001 |
| Daily cigarette use | - | | - | | - | | 0.94 (-0.30, 2.18) | .138 |
| Alcohol use >=2 days weekly | - | | - | | - | | -0.94 (-2.11, 0.23) | .115 |
| Other illicit drug use (>=one day per month | - | | - | | - | | 0.02 (-0.83, 0.88) | .960 |

Figure 1. Mean baseline CUDIT-R scores across DSM-CUD severity categories
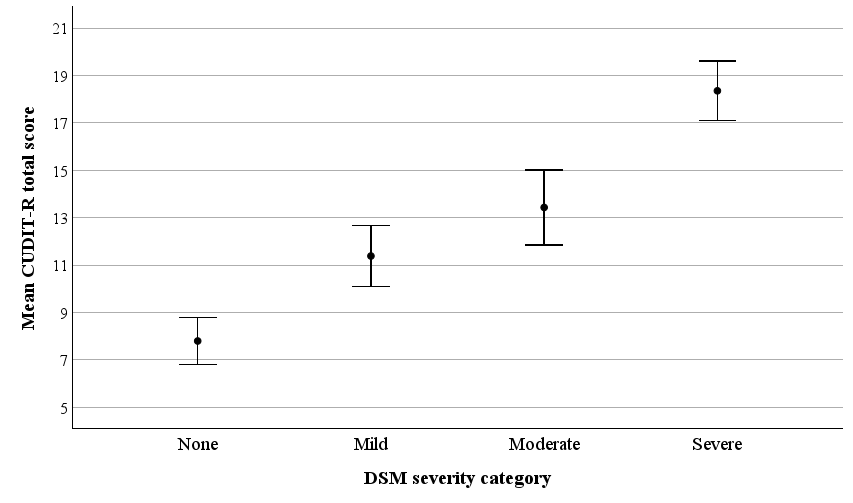
*Note: The CUDIT-R was assessed over the past 3 months, whereas the DSM interview assessed past-year experiences.
